# Whole genome short read data from 567 bulls of 14 breeds provides insight into genetic diversity of French cattle

**DOI:** 10.1016/j.dib.2025.112049

**Published:** 2025-09-09

**Authors:** Mekki Boussaha, Camille Eché, Christophe Klopp, Cécile Grohs, Marine Milhes, Amandine Suin, Tabatha Bulach, Rachel Fourdin, Thomas Faraut, Claire Kuchly, Sébastien Fritz, Caroline Vernette, Maulana Naji, Valentin Sorin, Aurélien Capitan, Christine Gaspin, Denis Milan, Didier Boichard, Carole Iampietro, Cécile Donnadieu

**Affiliations:** a Université Paris-Saclay, INRAE, AgroParisTech, GABI, 78350 Jouy-en-Josas, France; b INRAE, US 1426, GeT-PlaGe, Genotoul, Castanet-Tolosan, France; c Plateforme Bio-informatique Genotoul, Mathématiques et Informatique Appliquées de Toulouse, INRAE, Castanet-Tolosan, France; d GenPhySE, Université de Toulouse, INRAE, INPT, ENVT, Castanet-Tolosan Cedex, F-31326, France; e ELIANCE, 75012 Paris, France

**Keywords:** Sequencing, Variants, Bovine, Pangenome, Mutations, SNP, Polymorphism

## Abstract

Technological developments in high-throughput sequencing and advances in bioinformatic analysis allowed to sequence and study a very large number of genomes from a single species (cattle). Analyzing this data set enabled to generate the corresponding genomic variant database, especially for single nucleotide polymorphisms (SNPs) and small insertion or deletion (Indels) variations. These variants and genotypes allowed to better characterize the genetic diversity of these breeds.

In this work, we sequenced 567 bulls from 14 different breeds (Holstein, Montbéliarde, Normande, Brown Swiss, Simmental, Abondance, Tarentaise, Vosgienne, Blonde d'Aquitaine, Charolaise, Limousine, Aubrac, Flamande, Parthenaise). Each sample was sequenced at an approximately 15x depth on the Illumina Novaseq6000 platform. We detected 34,252,080 variants, 25,115,987 of which were already known in the Ensembl variation database version 110 and 9,136,093 were absent and were considered as novel variants. This data set represents a useful resource for the community to better identify SNPs or indels such as mutation anticipation and provides new insights into bovine genetic diversity.

Specifications Table

**Table:** 

Subject	Animal Science
Specific subject area	Investigating the genetic diversity of French cattle by sequencing the genomes of animals from multiple breeds and providing a broader range of SNPs.
Type of data	Raw sequencing reads (fastq files), variant (vcf file)
Data collection	The animals have been selected to cover the whole country of France and to belong to the 2 breeding types (dairy and suckling). All genomic DNAs were isolated from frozen semen samples using an automated DNA purification system. Whole genome short reads were produced using NovaSeq6000 (Illumina).
Data source location	Frozen semen samples were provided from various French breeding companies. Extracted ADN was stored at INRAE, Jouy-en-Josas, France.
Data accessibility	Repository name: European Nucleotide Archive (ENA) and https://entrepot.recherche.data.gouv.fr/ Data identification number: PRJEB64023 (ENA) Direct URL to data: https://doi.org/10.57745/BIFKZFhttps://www.ebi.ac.uk/ena/browser/view/PRJEB64023
Related research article	None

## 1 Value of the Data

• This data set is valuable, as it includes young and founder bulls from 14 bovine dairy and suckling breeds, well representing the current French cattle population.
• All these sequencing data are available and can be used by other researchers, for example to anticipate mutations that cause genetic diseases and to allow a systematic diagnosis of all sequenced bulls.
• They are also useful for the detection and analysis of zootechnical variants in cattle breeding, whether for dairy or suckling breeds.
• What’s more, this exhaustive catalog of bovine genomic polymorphisms provides us with a reference base for imputing variants in animals genotyped by low pass sequencing.

## 2 Background

France has the largest cattle population in Europe: 17 million heads. Behind this figure lies a large diversity of breeds, with around fifty breeds adapted to different environmental conditions and farming systems [1].

Today, almost half of the cattle breeds have a selection system identifying bulls whose genetic value is based on the performance of their daughters. In each generation, bulls with optimal genetic value are selected for artificial insemination (AI), facilitating the spread of genetic improvement. Therefore, the ancestral bulls of a breed (also called founders) represent a significant portion of its genetic diversity. As a result, their genomes are likely to carry genetic variants that explain the phenotypic variability within the breed, as well as deleterious recessive variants that will be spread until they become homozygous.

Since each breed corresponds to a small number of founders, sequencing the genome of these founder sires allows us to better characterize the extent of the genetic diversity within and between breeds.

In the present study we sequenced the genome of 567 AI bulls corresponding to the 14 most important French cattle breeds for which a selection program is currently underway.

## 3 Data Description

All read sets were sequenced with Illumina Novaseq6000 technology (Table 1).

**Table 1:** Read set general description.

Number of breeds	14
Number of samples	567
Number of files	2802
Total number of reads	257,526,959,592
Total number of nucleotides	38,552,948,856,468

The 567 samples belong to 14 breeds selected for this dataset (Table 2). Among these, five breeds (Holstein, Montebéliarde, Normande, Brown Swiss also named Brune and Simmental), already have a lot of founders data from sequencing efforts in France or in the 1000GB project international consortium. For these breeds, it was decided to sequence the genomes of 316 animals corresponding to the most common young sires recently marketed for AI.

**Table 2:** Selected breeds and number of animals.

Breed	Short name	Number of animals
Abondance	**ABO**	13
Aubrac	**AUB**	40
Blonde d’Aquitaine	**BAQ**	30
Brune	**BSW**	9
Charolaise	**CHA**	60
Holstein	**HOL**	145
Limousine	**LIM**	88
Montbéliarde	**MON**	91
Normande	**NMD**	63
Parthenaise	**PAR**	4
Rouge Flamande	**RDC**	5
Simmentale	**SIM**	5
Tarentaise	**TAR**	10
Vosgienne	**VOS**	4
Total		567

For the other nine breeds (Abondance, Tarentaise, Vosgienne, Blonde d’Aquitaine, Charolaise, Limousine, Aubrac, Rouge Flamande, and Parthenaise), we sequenced the genome of important founder bulls. These were in fact “old” bulls that had been used extensively in AI in France and were considered as strong contributors to the breed genetics, and for which semen doses were available as biological samples.

**Fig. 1:**
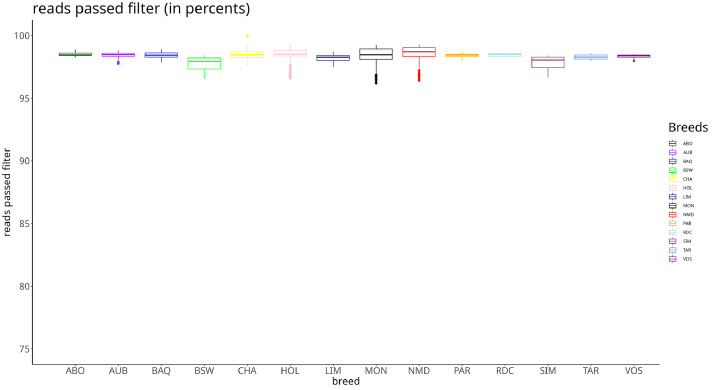
Distributions of read passing quality filters for each breed. The x axis presents the short names of different breeds (presented in Table 2) and the y axis the percentages of non-quality filtered reads. Each colored box plot presents the distribution of the percentage of non-quality filtered reads of the sampled bulls for a breed.

**Fig. 2:**
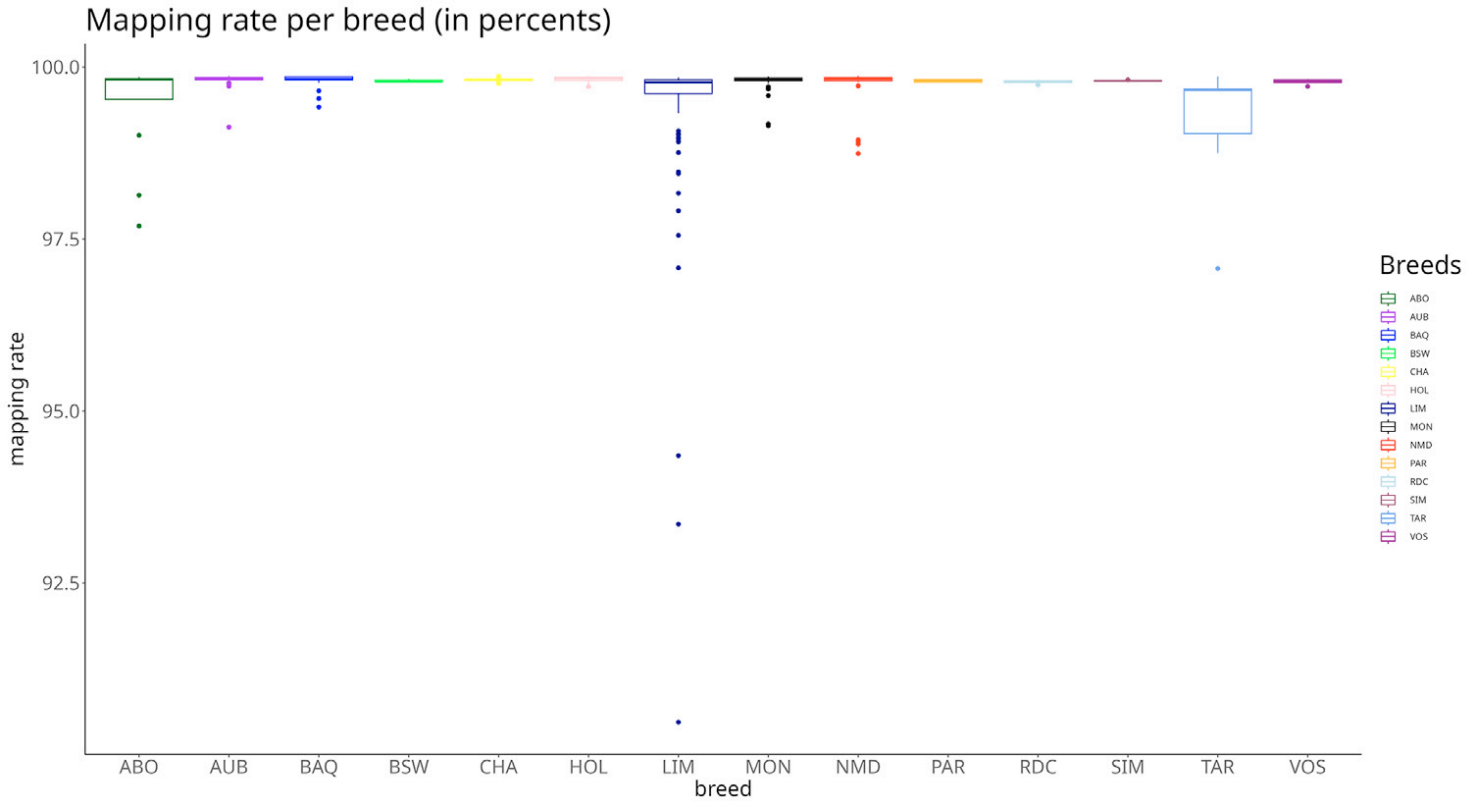
Mapping rate distribution for each breed. The x axis presents the short names of different breeds (presented in Table 2) and the y axis the percentage of reads mapped on the reference. Each colored box plot presents the distribution of the alignment rates of the bulls sampled for a breed.

**Fig. 3:**
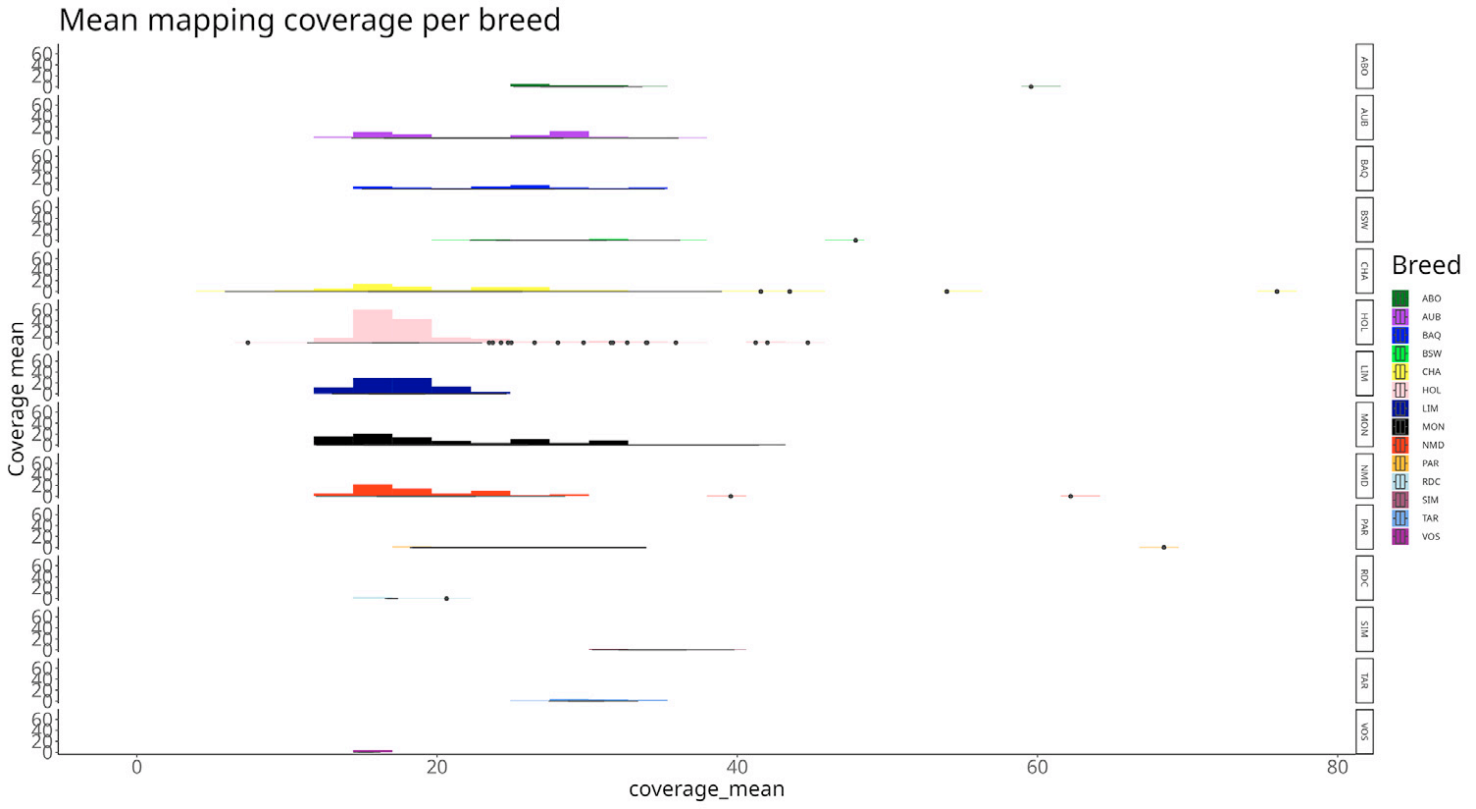
Read coverage for each breed. The x axis presents the reference genome coverage of each read bull read set. The y axis corresponds to the number of samples showing this coverage. Each horizontal block and color corresponds to a breed.

Proportion of read passing quality filters for all breeds was above 95 % (Fig. 1), and per breed mapping rate was above 90 % (Fig. 2). Average coverage for all breeds is at 15X (Fig. 3)

This sequence dataset allowed a search for small genome variations using the GATK-Unified Genotyper software. This resulted in the identification of 34,252,080 variants. Approximately 32,214,102 (94 %) were bi-allelic variants out of which 28,931,312 corresponded to SNPs, 1,836,784 were deletions and 1,446,006 insertions. For 12,516 insertions and 18,149 deletions, their size was higher than 50 nucleotides and can be categorized as structural variants. The largest deletions and insertions identified were respectively 308 and 289 bp long. Altogether, the variants covered the entire genome and on average we observed one polymorphism every 94 nucleotides (Table 3).

**Table 3:** Chromosomal distribution of variants. The columns correspond respectively to the chromosome Id, its length, the identified number of variants and the inter-variants distance which corresponds to the average distance on the reference genome between two variants (unit pb).

Chromosomes	Length	Variants	Distance between 2 variants on the reference genome
1	158,534,110	2,119,337	75
2	136,231,102	1,674,722	81
3	121,005,158	1,506,303	80
4	120,000,601	1,670,293	72
5	120,089,316	1,582,472	76
6	117,806,340	1,547,483	76
7	110,682,743	1,382,195	80
8	113,319,770	1,395,018	81
9	105,454,467	1,301,147	81
10	103,308,737	1,364,105	76
11	106,982,474	1,307,516	82
12	87,216,183	1,363,627	64
13	83,472,345	1,030,393	81
14	82,403,003	1,011,664	81
15	85,007,780	1,256,958	68
16	81,013,979	1,071,535	76
17	73,167,244	1,025,514	71
18	65,820,629	922,253	71
19	63,449,741	796,878	80
20	71,974,595	940,616	77
21	69,862,954	904,394	77
22	60,773,035	741,570	82
23	52,498,615	1,052,655	50
24	62,317,253	825,580	75
25	42,350,435	592,067	72
26	51,992,305	663,034	78
27	45,612,108	653,030	70
28	45,940,150	635,508	72
29	51,098,607	776,586	66
X	139,009,144	1,066,765	130
Y	43,300,181	70,862	611
Total	**2,628,394,924**	**34,252,080**	**94**

Overall, 25,115,987 (73.3 %) of the identified variants were known in the Ensembl variation 110 database (build 143). The remaining variants (∼27 %) were considered as novel variants and should help to better highlight the genetic variability in cattle. Out of these novel variants, 8,072,484 were found to be bi-allelic variants and corresponded to 6,083,529 SNPs, 764,692 small deletions, 15,670 large deletions (SVs), 1,198,685 small insertions and 9908 large insertions (SVs)

To evaluate the quality of our sequencing data-derived genotypes, we performed two different analyses.

First, we used the ratio of transitions over transversions (Ts/Tv) as a diagnostic measure to assess the quality of our sequencing data. The average Ts/Tv ratio observed in our whole-genome sequencing data was 2.26 and ranged from 2.24 to 2.28 (Table 4). This average rate is slightly higher than the 2.15 rate which we reported earlier [2]. In fact, it has been previously reported that the Ts/Tv ratio should be around 2.1 for whole-genome sequencing and that lower ratios may indicate that the sequencing data includes false positives caused by random sequencing errors [3]. Therefore, the Ts/Tv ratio estimated in our study is indicative of good sequencing data quality.

**Table 4:** Distribution of Ts/Tv (transitions / transversions).

Transitions	3,546,644,621
Transversions	1,569,468,314
Ts/Tv ratio	2.26

Note: Only SNPs are used for this statistic. This Ts/Tv ratio is a 'raw' ratio (ratio of observed events).

Second, we compared our sequencing data-derived genotypes to SNP array-derived genotypes using the Bovine 50 K SNP BeadChip (BovineSNP50 BeadChip version 1 or 2, Illumina, San Diego, CA). Overall, both sequence-derived and SNP assay produced genotyping data sources were available for 557 samples. The average genotype concordance rate was around 99.51 % and ranged from 94.63 to 99.85 % (see additional file “concordance_rate”).

**Fig. 4:**
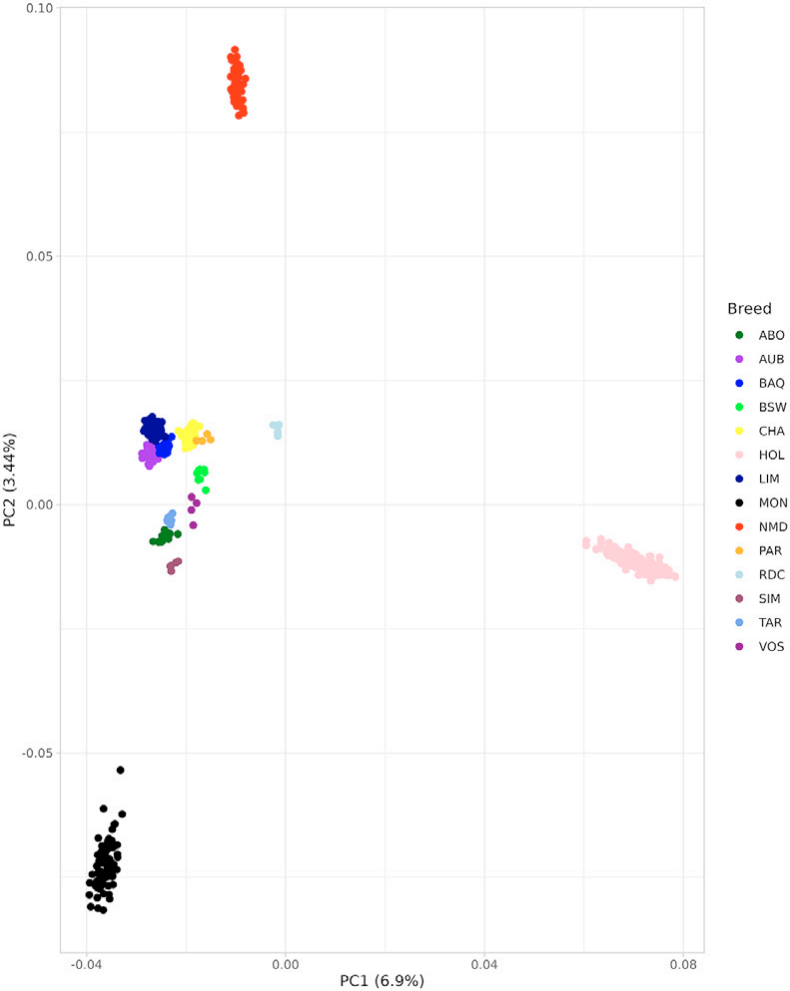
Results of PCA analysis. PCA (PC1/PC2) results were shown for the 14 breeds used in the study.

In order to validate the correct assignment of animals to their breed, we conducted PCA analysis. After applying all filters (described in the Materials and Methods section), 664,950 variants were retained and used for principal component analysis. Our PCA approach allowed us to describe the genetic structure of the 14 breeds and showed that all animals were successfully assigned to their corresponding breed and provided a good description of the breed structure (Fig. 4). Our results are of particular interest as they could be considered as a global statistical validation step to evaluate the quality of sequence-derived genotypes.

## 4 Experimental Design, Materials and Methods

### 4.1 Sample collection & technical validation

Semen samples were collected from ejaculates of selection program AI bulls by cattle breeding companies, and stored with solvent-free animal product. The Qiasymphony extraction kit was used to extract DNA from these samples. DNA was stored at −20 °C. Genomic DNA concentrations were measured using the Qubit fluorimetry system (Life Technologies) with the High Sensitivity (HS) kit for detection of double-stranded DNA (Thermo Fisher, Part #Q32854). Fragment size distributions were assessed using the Fragment Analyser Kit (Agilent). Purity was measured using a Nanodrop system (Thermofisher). All samples were purified with AMPureXP beads (Beckman Coulter, A63882) to obtain correct ratios i.e. 260/280: 1.8–2 and 260/230:2–2.2.

### 4.2 Sequencing

All sequencing processes were performed at the GeT-PlaGe core facility at INRAE Toulouse, France (DOI: 10.17180/nvxj-5333) (http://get.genotoul.fr).

DNA-seq libraries were prepared according to the Illumina protocol TruSeq NanoDNA. DNA was fragmented by sonication on Pixul, size selection was performed using Sample Purification Beads of the library kit (ratio 1/1 beads water) and adaptators IDT for Illumina – TruSeq DNA UD Indexes (96 Indexes, 96 Samples, 20,022,370) were ligated before sequencing. Library quality was assessed using a Fragment Analyzer (Agilent) with High Sensitivity NGS Kit (DNF-474–0500). Sizes of 800 bp were obtained. They were then quantified by qPCR using the KAPA Library Quantification Kit (Roche, 07,960,140,001) and sequenced by pools of ∼60. Pools were sequenced on 34 S4 lanes and 4 SP lanes NovaSeq 6000 using a paired-end read length of 2 × 150 bp with the Illumina Reagent kit (300 cycles) which corresponds to 19X coverage.

To evaluate the quality of our sequencing data-derived genotypes, we compared, for each sample, our sequencing data-derived genotypes to SNP array-derived genotypes using the Illumina 50 K Bovine SNP assay (BovineSNP50 BeadChip version 1 or 2, Illumina, San Diego, CA).

### 4.3 Whole-genome sequence alignment to the reference and variant calling

Raw paired read sequences were first trimmed of adapters, low quality bases (qscore <20) at start and end. Reads with mean qscore less than 20 or length less than 35 bp were then filtered out using the Trimmomatic software version 0.38 [4,5].

Sequence alignments were carried out using the Burrows-Wheeler Alignment tool (BWA 0.7.17 [6] with the mem option with default parameters for mapping reads to the ARS-UCD1.2 bovine reference genome. Potential PCR duplicates, which can adversely affect the variant calls, were removed using the MarkDuplicates tools from the Picard package version v2.18.2 [7]. Base quality recalibration was subsequently performed according to GATK best practices guidelines [8]. Only properly paired reads with a mapping quality of at least 30 (−*q* = 30) were retained. The resulting BAM files were then used to produce a GVCF file for each sample. Finally, variant calling was performed on all GVCF files using the GATK GenotypeGVCFs options.

### 4.4 Population Structure Analysis

We conducted PCA analysis using version 2.00a4 of the PLINK software [9], and used the ggplot2 library in R for visualizing the results [10]. First, we performed SNP quality control, defining our criteria as follows: only SNPs were used in the analysis, only autosomes were selected for further analysis, only call rates of more than 95 % were kept, and only minor allele frequency of more than 0.05 was accepted. Moreover, to avoid biases caused by linkage disequilibrium, we also pruned SNPs for LD using the command ‘indep (50 10 0.1)’. Finally, we calculated PCA (–pca 566) and visualized it by the ggplot2 package in R (V4.3.3).

## Limitations

Not applicable.

## Ethics Statement

The experiments reported in this paper comply with the ethical guidelines of the French National Research Institute for Agriculture, Food and Environment (INRAE) and its French research partners. No animal was intentionally bred for this study, and no invasive sampling was performed. Therefore, no ethical approval was required for this study. Breeding companies, as partners in the project, provided commercial straws. These samples were not produced for the project, but are prepared and distributed for AI as part of cattle selection programs.

## Credit Author Statement

**M Boussaha:** Formal analysis, Visualization, Validation, Writing - Original Draft, Writing - Review & Editing. **C Eché:** Writing - Original Draft, Investigation. **C Grohs:** Resources, Investigation, Writing - Original Draft, Writing - Review & Editing. **M Milhes:** Investigation. **A Suin:** Investigation. **T Bulach:** Investigation. **R Fourdin:** Investigation. **C Kuchly:** Data Curation. **C Klopp:** Formal analysis, Visualization, Validation, Writing - Original Draft, Writing - Review & Editing. **C Vernette:** Data Curation. **T Faraut:** Writing - Original Draft. **S Fritz:** Resources, Conceptualization. **M Naji:** Formal analysis, Visualization, Review & Editing. **V Sorin:** Formal analysis. **A Capitan:** Writing - Original Draft, Writing - Review & Editing. **D Boichard:** Funding acquisition, Conceptualization. **C Gaspin:** Funding acquisition, Conceptualization. **D Milan:** Funding acquisition, Conceptualization: Funding acquisition, Conceptualization. **C Iampietro:** Project administration, Supervision, Writing - Original Draft, Writing - Review & Editing. **C Donnadieu:** Funding acquisition, Conceptualization, Supervision.

## Funding

This work was supported by “La Région Occitanie” and 10.13039/100015221European Union (Operational Program FEDER-FSE MIDI-PYRENEES ET GARONNE 2014–2020, call for projects “Regional Platforms of Research and Innovation” of the Occitanie region). APIS-GENE and also industrial organisations of the ruminant sector CNIEL, Interbev, Eliance, CNE who contributed to the financing of these analyses. GeT-PlaGe facility was supported by France Génomique National infrastructure, funded as part of “Investissement d’avenir” program managed by 10.13039/501100005304Agence Nationale pour la Recherche (contract ANR-10-INBS-09).

## Data Availability

DataverseWhole-genome sequencing of 567 bulls (Original data)
DataverseGenetic variants from 567 French cattle samples (Original data)

## Footnotes

Supplementary data associated with this article can be found, in the online version, at doi:10.1016/j.dib.2025.112049.
