# Supplementary material for: Whole genome short read data from 567 bulls of 14 breeds provides insight into genetic diversity of French cattle
Source: Data Brief. 2025 Sep 9;62:112049. doi: 10.1016/j.dib.2025.112049 (PMC12481132; doi:10.1016/j.dib.2025.112049)
Supplement: Supplementary file 1 [file mmc1.pdf]

Breed	Sample ID	Concordance rate
ABO	40740	0.9937
ABO	40741	0.9934
ABO	40742	0.979
ABO	40743	0.9979
ABO	40744	0.9919
ABO	40745	0.9956
ABO	40746	0.9973
ABO	40747	0.9944
ABO	40748	0.9928
ABO	40749	0.999
ABO	40750	0.9963
ABO	40751	0.9981
ABO	40752	0.9971
AUB	40753	0.9928
AUB	40754	0.9945
AUB	40755	0.9949
AUB	40756	0.9956
AUB	40757	0.9977
AUB	40758	0.9897
AUB	40759	0.9944
AUB	40760	0.9933
AUB	40761	0.9944
AUB	40762	0.9957
AUB	40763	0.9954
AUB	40764	0.9953
AUB	40765	0.9964
AUB	40766	0.9937
AUB	40767	0.9971
AUB	40768	0.9958
AUB	40769	0.9479
AUB	40770	0.98
AUB	40771	0.9847
AUB	40772	0.9956
AUB	41132	0.9951
AUB	41133	0.9967
AUB	41134	0.9919
AUB	41135	0.9931
AUB	41136	0.9952
AUB	41137	0.9962
AUB	41138	0.9964
AUB	41139	0.9927
AUB	41140	0.9947
AUB	41141	0.9976
AUB	41142	0.9907
AUB	41143	0.9977
AUB	41144	0.994
AUB	41145	0.9972
AUB	41146	0.9948
AUB	41147	0.9978

AUB	41148	0.9964
AUB	41149	0.9945
AUB	41150	0.9942
AUB	41151	0.9932
BAQ	40783	0.9964
BAQ	40784	0.994
BAQ	40785	0.9939
BAQ	40786	0.9963
BAQ	40787	0.9948
BAQ	40788	0.9947
BAQ	40789	0.9958
BAQ	40790	0.9923
BAQ	40791	0.9926
BAQ	40792	0.9949
BAQ	40793	0.9937
BAQ	40795	0.991
BAQ	40796	0.9965
BAQ	40797	0.9947
BAQ	40798	0.9909
BAQ	40799	0.9889
BAQ	40800	0.9923
BAQ	40801	0.9924
BAQ	40802	0.9976
BAQ	40803	0.9969
BAQ	41152	0.9971
BAQ	41153	0.9972
BAQ	41154	0.9958
BAQ	41155	0.9984
BAQ	41156	0.9943
BAQ	41157	0.9965
BAQ	41158	0.9967
BAQ	41159	0.9978
BAQ	41160	0.9967
BAQ	41161	0.9969
BSW	40977	0.9985
BSW	40978	0.9862
BSW	40979	0.9983
BSW	40980	0.9984
BSW	40981	0.999
BSW	40982	0.9984
BSW	40983	0.9987
BSW	40984	0.9989
BSW	40985	0.999
CHA	41056	0.9988
CHA	41057	0.9982
CHA	41058	0.9463
CHA	41059	0.9942
CHA	41060	0.9974
CHA	41061	0.9987
CHA	41062	0.9979

CHA	41063	0.9915
CHA	41064	0.9988
CHA	41065	0.9987
CHA	41066	0.9974
CHA	41067	0.9972
CHA	41068	0.9962
CHA	41069	0.9983
CHA	41070	0.998
CHA	41071	0.9991
CHA	41072	0.999
CHA	41073	0.9957
CHA	41074	0.997
CHA	41075	0.9904
CHA	41076	0.9931
CHA	41077	0.988
CHA	41078	0.9983
CHA	41079	0.9978
CHA	41080	0.9985
CHA	41081	0.9988
CHA	41082	0.9974
CHA	41083	0.9871
CHA	41084	0.9927
CHA	41085	0.9948
CHA	41086	0.9927
CHA	41087	0.992
CHA	41088	0.996
CHA	41089	0.9933
CHA	41090	0.997
CHA	41091	0.9944
CHA	41092	0.9923
CHA	41162	0.9982
CHA	41163	0.9975
CHA	41164	0.9957
CHA	41165	0.9962
CHA	41166	0.9884
CHA	41167	0.9974
CHA	41168	0.9964
CHA	41169	0.9951
CHA	41170	0.9957
CHA	41171	0.9985
CHA	41172	0.9958
CHA	41173	0.9936
CHA	41174	0.989
CHA	41175	0.9968
CHA	41176	0.9966
CHA	41177	0.979
CHA	41178	0.9918
CHA	41179	0.9963
CHA	41180	0.9936
CHA	41181	0.9836

CHA	41183	0.9975
CHA	41184	0.9922
HOL	40532	0.9983
HOL	40533	0.998
HOL	40534	0.9971
HOL	40535	0.9911
HOL	40536	0.9975
HOL	40537	0.9973
HOL	40538	0.9982
HOL	40539	0.9974
HOL	40540	0.9971
HOL	40541	0.9965
HOL	40542	0.9988
HOL	40543	0.9957
HOL	40544	0.9954
HOL	40545	0.99
HOL	40546	0.9951
HOL	40547	0.9929
HOL	40548	0.9833
HOL	40549	0.9979
HOL	40550	0.9978
HOL	40551	0.9987
HOL	40555	0.9974
HOL	40556	0.9946
HOL	40557	0.9947
HOL	40558	0.9912
HOL	40559	0.9978
HOL	40560	0.9975
HOL	40561	0.9985
HOL	40562	0.987
HOL	40563	0.9846
HOL	40564	0.9932
HOL	40565	0.9816
HOL	40566	0.9917
HOL	40567	0.9899
HOL	40568	0.9822
HOL	40569	0.9947
HOL	40570	0.9934
HOL	40571	0.9915
HOL	40572	0.9907
HOL	40573	0.9848
HOL	40574	0.9875
HOL	40575	0.9878
HOL	40576	0.9912
HOL	40577	0.9964
HOL	40578	0.9987
HOL	40579	0.9947
HOL	40669	0.993
HOL	40670	0.9949
HOL	40671	0.9971

HOL	40672	0.9958
HOL	40673	0.9958
HOL	40674	0.9966
HOL	40675	0.9963
HOL	40676	0.9987
HOL	40677	0.9969
HOL	40678	0.9989
HOL	40679	0.9983
HOL	40680	0.9977
HOL	40681	0.9984
HOL	40682	0.9943
HOL	40683	0.9977
HOL	40684	0.9974
HOL	40685	0.9956
HOL	40686	0.9984
HOL	40687	0.9944
HOL	40688	0.9877
HOL	40689	0.9963
HOL	40690	0.9947
HOL	40691	0.9964
HOL	40692	0.9913
HOL	40693	0.9954
HOL	40694	0.9973
HOL	40695	0.9946
HOL	40696	0.9892
HOL	40697	0.9892
HOL	40698	0.9955
HOL	40699	0.994
HOL	40700	0.9981
HOL	40701	0.9969
HOL	40702	0.9979
HOL	40703	0.9969
HOL	40704	0.9936
HOL	40705	0.9947
HOL	40706	0.9962
HOL	40707	0.9952
HOL	40708	0.9983
HOL	40709	0.9964
HOL	40710	0.9944
HOL	40711	0.9938
HOL	40712	0.9961
HOL	40713	0.9963
HOL	40714	0.9952
HOL	40715	0.994
HOL	40716	0.9923
HOL	40717	0.9959
HOL	40718	0.9694
HOL	41011	0.9987
HOL	41012	0.9989
HOL	41013	0.999

HOL	41014	0.9992
HOL	41015	0.999
HOL	41016	0.9989
HOL	41017	0.999
HOL	41018	0.9988
HOL	41019	0.999
HOL	41020	0.9989
HOL	41021	0.9938
HOL	41022	0.9985
HOL	41023	0.998
HOL	41024	0.9978
HOL	41025	0.9956
HOL	41026	0.9946
HOL	41027	0.9964
HOL	41028	0.998
HOL	41029	0.9953
HOL	41030	0.9975
HOL	41031	0.9984
HOL	41032	0.9977
HOL	41033	0.9989
HOL	41034	0.997
HOL	41189	0.9971
HOL	41190	0.9955
HOL	41191	0.9946
HOL	41192	0.992
HOL	41193	0.9949
HOL	41194	0.9925
HOL	41195	0.9943
HOL	41196	0.992
HOL	41197	0.9959
HOL	41198	0.9927
HOL	41199	0.9943
HOL	41200	0.9962
HOL	41201	0.9964
HOL	41202	0.9953
HOL	41203	0.9984
HOL	41204	0.9955
HOL	41205	0.9968
HOL	41206	0.9913
HOL	41207	0.9967
HOL	41208	0.9955
HOL	41209	0.9966
HOL	41210	0.9956
HOL	41211	0.9944
HOL	41212	0.9953
HOL	41213	0.9948
LIM	41219	0.9791
LIM	41220	0.9964
LIM	41221	0.9968
LIM	41222	0.997

LIM	41223	0.9961
LIM	41224	0.9935
LIM	41225	0.9953
LIM	41226	0.9926
LIM	41227	0.9732
LIM	41228	0.9914
LIM	41229	0.997
LIM	41230	0.9935
LIM	41231	0.9969
LIM	41232	0.9958
LIM	41233	0.996
LIM	41234	0.995
LIM	41235	0.9955
LIM	41236	0.9947
LIM	41237	0.9978
LIM	41238	0.9941
LIM	41239	0.9972
LIM	41241	0.9962
LIM	41242	0.9947
LIM	41243	0.9941
LIM	41244	0.9938
LIM	41245	0.9956
LIM	41246	0.9935
LIM	41247	0.9892
LIM	41248	0.9982
LIM	41249	0.9975
LIM	41250	0.9956
LIM	41252	0.9951
LIM	41253	0.9943
LIM	41254	0.9914
LIM	41255	0.9903
LIM	41256	0.9896
LIM	41257	0.9943
LIM	41258	0.9964
LIM	41259	0.9965
LIM	41260	0.9903
LIM	41261	0.9981
LIM	41262	0.9893
LIM	41263	0.9977
LIM	41264	0.9946
LIM	41265	0.9971
LIM	41266	0.9942
LIM	41268	0.9878
LIM	41269	0.9987
LIM	41270	0.9899
LIM	41272	0.9884
LIM	41273	0.9923
LIM	41274	0.9972
LIM	41275	0.997
LIM	41276	0.9876

LIM	41277	0.9945
LIM	41278	0.9944
LIM	41279	0.9915
LIM	41280	0.9917
LIM	41281	0.9972
LIM	41283	0.9859
LIM	41284	0.9981
LIM	41285	0.9981
LIM	41286	0.9768
LIM	41287	0.9984
LIM	41288	0.997
LIM	41289	0.997
LIM	41290	0.9982
LIM	41291	0.9977
LIM	41292	0.996
LIM	41293	0.9988
LIM	41294	0.9969
LIM	41295	0.9949
LIM	41296	0.9943
LIM	41297	0.992
LIM	41298	0.9952
LIM	41299	0.9983
LIM	41300	0.9972
LIM	41301	0.9975
LIM	41302	0.9964
LIM	41303	0.9983
LIM	41304	0.9953
LIM	41305	0.9956
LIM	41306	0.9956
MON	40492	0.9975
MON	40493	0.9954
MON	40494	0.9971
MON	40495	0.9956
MON	40496	0.9935
MON	40497	0.9934
MON	40498	0.9963
MON	40499	0.9968
MON	40500	0.9962
MON	40501	0.9957
MON	40502	0.9903
MON	40503	0.9943
MON	40504	0.9956
MON	40505	0.9952
MON	40506	0.9954
MON	40507	0.9948
MON	40508	0.9963
MON	40509	0.9948
MON	40510	0.9958
MON	40511	0.9943
MON	40600	0.9973

MON	40601	0.9979
MON	40602	0.9982
MON	40603	0.9964
MON	40604	0.9975
MON	40605	0.9982
MON	40606	0.9969
MON	40607	0.9968
MON	40608	0.9807
MON	40609	0.9963
MON	40610	0.9941
MON	40611	0.9989
MON	40612	0.9978
MON	40613	0.9972
MON	40614	0.9964
MON	40615	0.9972
MON	40616	0.9974
MON	40617	0.9972
MON	40618	0.9962
MON	40619	0.9946
MON	40620	0.998
MON	40621	0.9979
MON	40622	0.9978
MON	40623	0.9979
MON	40624	0.9986
MON	40625	0.9938
MON	40626	0.9954
MON	40627	0.9955
MON	40628	0.994
MON	40629	0.9963
MON	40630	0.9859
MON	40631	0.9975
MON	40632	0.9966
MON	40633	0.9982
MON	40634	0.9934
MON	40635	0.9958
MON	40636	0.9971
MON	40637	0.9949
MON	40638	0.9978
MON	40639	0.9947
MON	40640	0.9964
MON	40968	0.9978
MON	40969	0.9985
MON	40970	0.9984
MON	40971	0.9985
MON	40972	0.9987
MON	40973	0.9985
MON	40974	0.9981
MON	40975	0.9987
MON	40976	0.9986
MON	40991	0.999

MON	40992	0.9984
MON	40993	0.9992
MON	40994	0.9988
MON	40995	0.9988
MON	40996	0.9989
MON	40997	0.9987
MON	40998	0.9988
MON	40999	0.9989
MON	41000	0.998
MON	41001	0.9988
MON	41002	0.9987
MON	41003	0.9988
MON	41004	0.9983
MON	41005	0.9986
MON	41006	0.9987
MON	41007	0.9982
MON	41008	0.9988
MON	41009	0.9986
MON	41010	0.9983
NMD	40512	0.997
NMD	40513	0.9918
NMD	40514	0.9959
NMD	40515	0.9957
NMD	40516	0.9915
NMD	40517	0.9967
NMD	40518	0.9958
NMD	40519	0.9958
NMD	40520	0.9966
NMD	40521	0.9979
NMD	40522	0.998
NMD	40523	0.9965
NMD	40524	0.9987
NMD	40525	0.9957
NMD	40526	0.9975
NMD	40527	0.9971
NMD	40528	0.9969
NMD	40529	0.9893
NMD	40530	0.9969
NMD	40531	0.9965
NMD	40580	0.997
NMD	40581	0.9952
NMD	40582	0.9961
NMD	40583	0.9966
NMD	40584	0.9962
NMD	40586	0.9948
NMD	40587	0.9944
NMD	40588	0.9945
NMD	40589	0.9967
NMD	40590	0.9983
NMD	40591	0.9941

NMD	40592	0.9977
NMD	40593	0.9986
NMD	40594	0.9954
NMD	40595	0.9942
NMD	40596	0.9975
NMD	40597	0.997
NMD	40598	0.998
NMD	40599	0.997
NMD	40960	0.9986
NMD	40961	0.9982
NMD	40962	0.9986
NMD	40963	0.998
NMD	40964	0.9989
NMD	40965	0.9983
NMD	40966	0.9947
NMD	41035	0.9984
NMD	41036	0.9983
NMD	41037	0.9983
NMD	41038	0.995
NMD	41039	0.998
NMD	41040	0.9983
NMD	41041	0.9978
NMD	41042	0.9985
NMD	41043	0.9983
NMD	41044	0.9968
NMD	41045	0.994
NMD	41046	0.9982
NMD	41047	0.9986
NMD	41048	0.9832
NMD	41049	0.9911
NMD	41050	0.9988
NMD	41051	0.9984
PAR	41052	0.9959
PAR	41053	0.9963
PAR	41054	0.9979
PAR	41055	0.9987
RDC	41214	0.9936
RDC	41215	0.9931
RDC	41216	0.9936
RDC	41217	0.9954
SIM	40986	0.9991
SIM	40987	0.9991
SIM	40988	0.9989
SIM	40989	0.9988
SIM	40990	0.999
TAR	40774	0.996
TAR	40775	0.9949
TAR	40776	0.9948
TAR	40777	0.9947
TAR	40778	0.9944

TAR	40779	0.9973
TAR	40780	0.9959
TAR	40781	0.9915
TAR	40782	0.996
VOS	41185	0.9878
VOS	41186	0.9953
VOS	41187	0.9962
VOS	41188	0.9931
